# The Role and Application of Exosomes in Gastric and Colorectal Cancer

**DOI:** 10.3389/fphar.2021.825475

**Published:** 2022-01-17

**Authors:** Qirong Li, Dongxu Wang, Dayong Ding, Ye Feng, Ruizhi Hou, Dianfeng Liu, Chao Lin, Yongjian Gao

**Affiliations:** ^1^ Department of Gastrointestinal Colorectal and Anal Surgery, China-Japan Union Hospital of Jilin University, Changchun, China; ^2^ Laboratory Animal Center, College of Animal Science, Jilin University, Changchun, China; ^3^ School of Grain Science and Technology, Jilin Business and Technology College, Changchun, China

**Keywords:** gastric cancer, colorectal cancer, ncRNA, exosomes, cancer therapy

## Abstract

Gastric cancer and colorectal cancer are malignant tumors found in the human gastrointestinal tract. Bidirectional communication between tumor cells and their microenvironment can be realized through the transmission of exosomes—small, cell-derived vesicles containing complex RNA and proteins. Exosomes play an important role in the proliferation, metastasis, immune response, and drug resistance of cancer cells. In this review, we focus on the role and application of exosomes in gastric and colorectal cancer. We also summarize the role of exosomes secreted by different types of cells in tumor development and as drug carriers in cancer treatment.

## Introduction

Gastric and colorectal cancer are globally-important diseases with high molecular and phenotypic heterogeneity (Smyth et al., 2020). Gastric cancer can be caused by a variety of genetic and epigenetic mutations, and *Helicobacter pylori* is also an important pathogenic factor (Uemura et al., 2001). The tumor microenvironment has a strong influence on the survival and treatment response of gastric cancer patients (Quail and Joyce, 2013). At present, early diagnosis of gastric cancer remains problematic because clinical symptoms often only appear in the late stages of cancer development, which significantly limits treatment options (Maconi et al., 2008). Colorectal cancer is the fourth most deadly cancer in the world; its etiologies include eating habits, old age, and smoking (Dekker et al., 2019). Colorectal cancer is normally treated with adjuvant therapy after surgical resection. However, the risk of subsequent cancer recurrence and metastasis remains high, and it is often related to resistance to traditional therapies such as chemotherapy and radiation (Jänne and Mayer, 2000). As gastric and colorectal cancers are associated with high morbidity and mortality, research into new targeted therapies is urgent.

**FIGURE 1:**
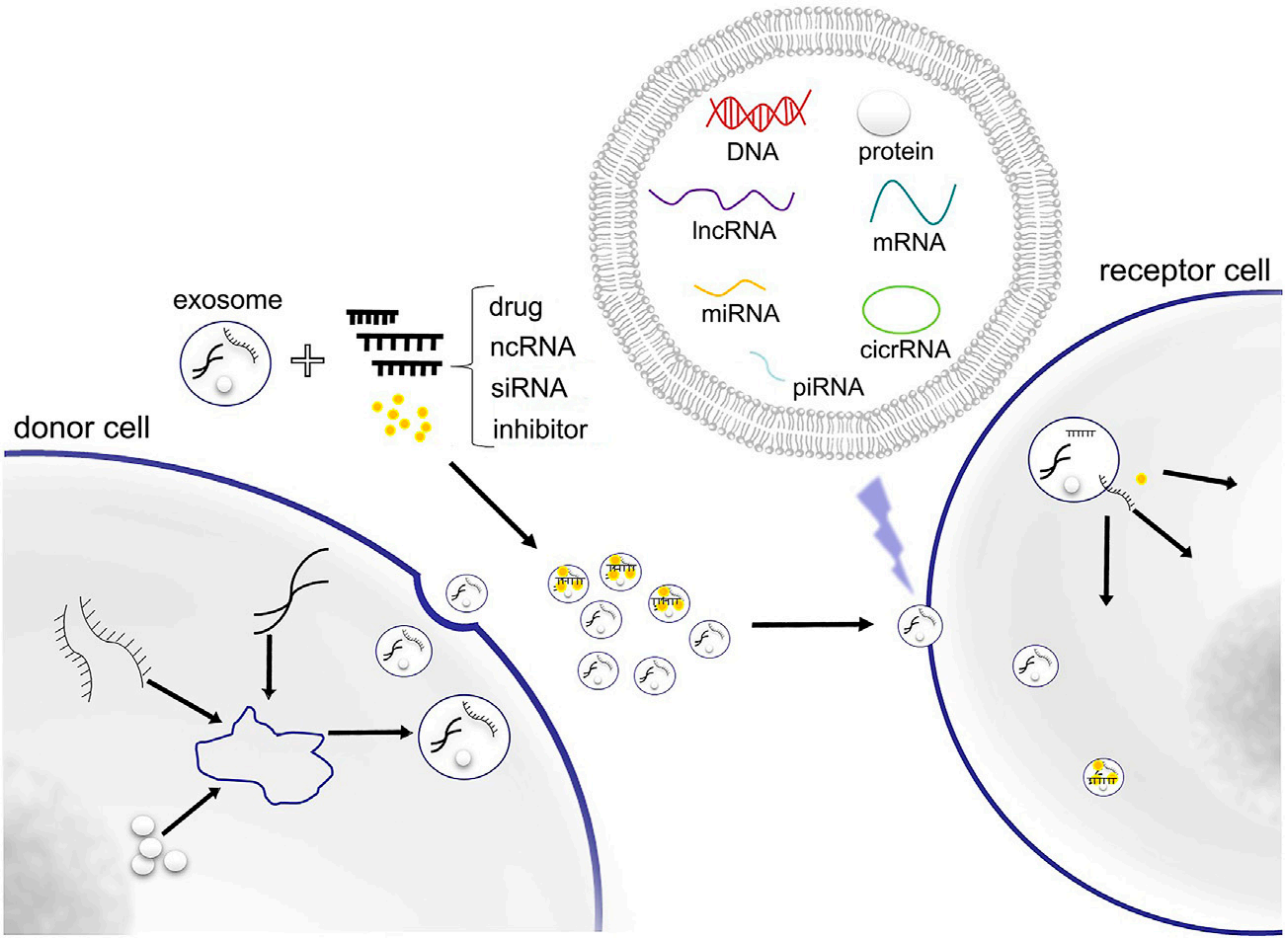
Exosome contains DNA, RNA, protein and other substances for cellular component communication. Exosomes can also be used as delivery vectors of drugs, ncRNA, siRNA and inhibitor.

Recent studies have shown that exosomes can be used as targeted drug carriers. Exosomes are small endocytic vesicles secreted by most cells (Théry et al., 2002), the diameters of which range between 40 and 100 nm. Exosomes have been found to be able to deliver bioactive molecules or other substances to specific recipient cells for intercellular communication (Figure 1). Increasing numbers of studies have indicated that exosomes are important nanomaterials that can regulate essential biological behaviors through intercellular transmission (Yang et al., 2019). They also play an important role in the pathogenesis of many diseases, including cancer, as they are involved in the apoptosis of tumor cells, the proliferation and migration of cancer cells, regulation of the tumor microenvironment, and angiogenesis (Nabariya et al., 2020). Because of these characteristics, exosomes can also be used as an efficient targeted drug delivery system in cancer treatment.

## Regulation of Exosomes Derived From Cancer Cells on Gastric Cancer and Colorectal Cancer

Exosomes play an important role in the communication between tumor cells themselves and between the tumor and its microenvironment. Exosomes can transfer many types of biomolecules, including DNA, RNA, and protein. Exosomes derived from cancer cells affect the biological characteristics of recipient cells and change the tumor microenvironment by transmitting these bioactive molecules to regulate the tumor’s development process. Noncoding RNA (ncRNA), including microRNA, circRNA, and lncRNA, is one of the most important bioactive molecules which does not encode protein and perform their biological functions at the RNA level.

Studies have shown that miR-15b-3p is highly expressed in exosomes secreted by gastric cancer cells and miR-15b-3p can be transferred by exosomes to enhance migration, invasion, and proliferation; it also inhibit the apoptosis of gastric cancer through the DYNLT1/*caspase-3*/*caspase-9* signaling pathway (Wei et al., 2020). Exosomal miR-25-3p from colorectal cancer promotes cancer development by inducing vascular permeability and angiogenesis (Zeng et al., 2018). In addition, exosomal circSHKBP1 regulates the miR-582-3p/HUR/VEGF pathway, thereby promoting the progression of gastric cancer (Xie M. et al., 2020). Studies have also shown that exosomal lncRNA *91H* promotes the proliferation of colorectal cancer by changing HNRNPK expression (Gao et al., 2018).

Exosomes also deliver cancer-related molecules that can influence the chemotherapeutic resistance of recipient cells. Cisplatin is one of the most effective and commonly used of the basic chemotherapy drugs for advanced gastric cancer treatment (Keehn and Higgins, 1981). Studies have shown that exosomes from cisplatin-resistant gastric cancer can enhance gastric cancer cells resistance to cisplatin by delivering miR-500a-3p. Therefore, exosomal miR-500a-3p could potentially be used to predict and eliminate chemotherapy-resistant gastric cancer cells (Lin et al., 2020). Research has also shown that p-STAT3-containing exosomes contribute to 5-FU resistance in colorectal cancer cells (Zhang et al., 2019).

Protein is an important component of exosomes, and exosomes have been shown to regulate the liver microenvironment by delivering EGFR to promote liver metastasis of gastric cancer (Zhang et al., 2017). Furthermore, exosomal ANGPTL1 has been shown to alleviate liver metastasis in colorectal cancer by reprogramming Kupffer cells and reducing MMP9 expression (Jiang et al., 2021).

In addition to acting as cell-to-cell transporters, cancer-derived exosomes have also been widely used as biomarkers in cancer diagnosis. Tumors of unknown primary origin can be classified by extracting tumor-type specific proteins that are contained in exosomes from tissues and plasma. Therefore, exosomal proteins can be used as reliable biomarkers for cancer detection and classification (Hoshino et al., 2020). Other research has revealed that exosomal piRNAs in serum may be a biomarker for the diagnosis and monitoring of gastric cancer metastasis (Ge et al., 2020). LncRNA *HOTTIP* has been found to be significantly up-regulated in gastric cancer cell exosomes; it could be used as a new biomarker for determining the diagnosis and prognosis of gastric cancer (Zhao et al., 2018). Further to this, studies have indicated that exosomal circRNA-PNN is a potential biomarker of colorectal cancer (Xie Y. et al., 2020).

These results suggest that tumor-derived exosomes can promote cancer progression and influence drug resistance through ncRNA transmission. Moreover, tumor-derived exosomes can be used as biomarkers for cancer; this indicates that tumor-derived exosomes may be used to diagnose and treat gastric and colorectal cancer.

## Regulation of Exosomes From Other Cells on Gastric Cancer and Colorectal Cancer

**FIGURE 2:**
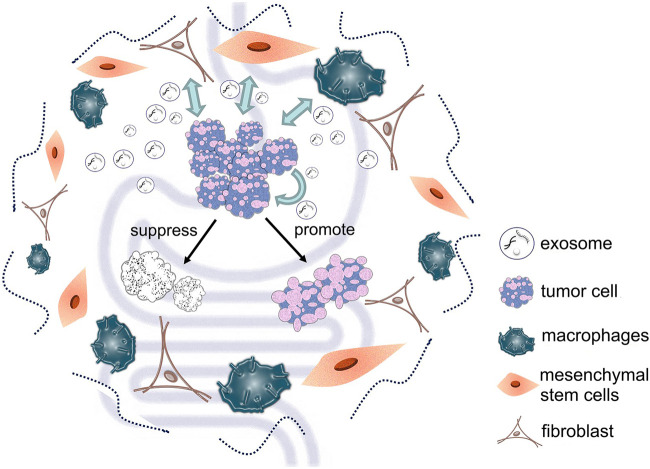
Different types of cell-derived exosomes in the tumor microenvironment can deliver various substances to tumor cells, thereby affecting tumor cell development.

Exosomes derived from cancer cells play an important role in intracellular communication, treatment and diagnosis of gastric and colorectal cancers. Similarly, other types of cell-derived exosomes in the tumor microenvironment are important for cancer progression (Figure 2). Cancer-associated fibroblasts (CAFs) are the main components of tumor stroma; they can influence tumor development and drug resistance by secreting exosomes. Studies have shown that exosomal miR-139 from CAFs can inhibit the progression and metastasis of gastric cancer by reducing MMP11 (Xu et al., 2019). Furthermore, miR-34 in exosomes secreted by CAFs can inhibit gastric cancer cell proliferation and invasion both *in vivo* and *in vitro* (Shi et al., 2020). Other research has found that exosomes delivery of miR-590-3p by CAFs can improve radioresistance of colorectal cancer by regulating the CLCA4-dependent PI3K/Akt signaling pathway (Chen X. et al., 2021). Moreover, CAFs can also enhance colorectal cancer drug resistance through exosomes delivery of lncRNA *H19* (Ren et al., 2018).

Tumor-associated macrophages (TAMs) are macrophages that infiltrate tumor tissue; they are the most abundant immune cells within the tumor microenvironment (Zhang Y. et al., 2018). Studies have shown that exosomes down-regulate PTEN and inhibit apoptosis by delivering TAMs-derived miR-21; they also improve cisplatin resistance in gastric cancer cells (Zheng et al., 2017). Research has also indicated that exosomes derived from M1 macrophages carry miR-16-5p; this activates the T-cell immune response through PD-L1 and inhibits gastric cancer proliferation (Li et al., 2020). In addition, exosomes derived from mouse TAMs have been shown to be associated with Th1/M1 polarization, inflammation, and enhancement of immune response in colorectal tumors (Cianciaruso et al., 2019). In M2 macrophage-derived exosomes, miR-21-5p and miR-155-5p were highly expressed, which mediated the migration and invasion of colorectal cancer cells (Lan et al., 2019).

Mesenchymal stem cells (MSCs) have the ability to self-replicate and they have strong differentiation potential, which means they can contribute to the formation of the tumor microenvironment and they can interact with cancer cells (Poggi and Giuliani, 2016). Exosomes derived from human umbilical cord mesenchymal stem cells (hUMSCs) that contain miR-6785-5p inhibit both gastric cancer angiogenesis and metastasis by inhibiting INHBA expression (Chen Z. et al., 2021). Moreover, exosomes derived from MSCs in gastric cancer tissues can deliver miR-221 to gastric cancer cells, thereby promoting their proliferation and migration (Wang et al., 2014). Studies have revealed that GARP knockdown MSCs inhibit the proliferation and invasion of mouse colorectal cancer cells through exosomes (Xing et al., 2020). It has also been shown that exosomes from bone marrow-derived mesenchymal stem cells (BMSCs) can promote stem cell-like characteristics of colorectal cancer through miR-142-3p (Li and Li, 2018).

Proteins delivered by CAFs, TAMs, and MSCs through exosomes are also important mediators of tumor and tumor microenvironment regulation. Studies that used proteomic analysis of CAFs and serum-derived exosomes have identified QSOX1 as a marker for non-invasive colorectal cancer detection (Ganig et al., 2021). TAM-derived exosomes have also been found to promote migration of gastric cancer cells by delivering functional apolipoprotein E (Zheng et al., 2018), and BMSCs exosomes with p53 deletion can regulate the Wnt/β-catenin pathway by transferring UBR2 to target cells, which promotes the growth and metastasis of gastric cancer (Mao et al., 2017). As can be seen from the above studies, CAFs, TAMs, and MSCs are important components of the tumor microenvironment; they cooperate with the cancer cells to regulate the tumor’s growth environment.

## Therapeutic Effects of Exosome-Carrying Drugs on Gastric Cancer

Recent studies have indicated that exosomes are the promising carrier of anticancer drugs and that exosome-based therapy may be an efficient and beneficial approach during cancer treatment. Exosomes have good biocompatibility, specificity, and low immunogenicity, and since they can be easily internalized by cells, they can effectively deliver drugs to cancer cells. The proteins and lipid compositions of tumor-derived exosomes are similar to those of the cells that secret them. (Sun W. et al., 2018), and report shows that exosomes from cancer cells are specifically taken up by the same type of tumor tissue or cell. If the tumor-derived exosomes are injected into the body, the exosomes will eventually return to the original tumor tissue. The tumor-targeting ability of tumor cell-derived exosomes may be related to the expression of integrin (Qiao et al., 2020). Because of this characteristic, tumor-derived exosomes are natural vectors that have great potential for targeted delivery of antitumor drugs. Studies have constructed nano aspirin exosome drug delivery systems that can effectively deliver aspirin to the tumor site *in vivo*; this induces apoptosis and autophagy of colorectal cancer cells, thus producing a good tumor treatment effect (Tran et al., 2019). In other study, exosomes were isolated from A33-positive LIM1215 cells and loaded with doxorubicin (DOX) to target A33-positive colorectal cancer cells. The results revealed that DOX-loaded exosomes have good tumor-targeting ability; they could also inhibit colorectal cancer growth (Li et al., 2018). In addition, researchers have loaded DOX into exosomes secreted by MSCs, which effectively inhibited colorectal tumor growth (Bagheri et al., 2020). Furthermore, there is evidence shown that exosomes coated with oxaliplatin and PGM5-AS1 can reverse drug resistance in colorectal cancer cells and inhibit their growth (Hui et al., 2021).

In addition to being used to package drugs, exosomes can also be used to deliver biological molecules for RNA-based therapeutic strategies. Hepatocyte growth factor (HGF) can promote the growth of tumor cells and blood vessels. HGF siRNA can be transported to cancer cells using exosomes, which can down-regulate expression of HGF, thereby inhibiting the proliferation and migration of gastric cancer cells (Zhang H. et al., 2018). Furthermore, exosomes can be used as nanoparticles to deliver anti-miR-214 and down-regulate the expression of miR-214 in gastric cancer cells, which can reduce cisplatin chemotherapy resistance in gastric cancer and inhibit tumor growth (Wang et al., 2018). Other research has shown that delivering c-Met siRNA via exosomes can promote cell apoptosis, inhibit tumor growth *in vivo*, and reduce gastric cancer cisplatin resistance (Zhang et al., 2020). It has also been reported that exosomes can be used to simultaneously deliver 5-FU and miR-21 inhibitors to colorectal cancer cells; researchers found that the constructed exosomes had significant anti-tumor effects both *in vivo* and *in vitro* (Liang et al., 2020). Furthermore, exosomes loaded with miR-128-3p from normal intestinal FHC cells have been found to be able to inhibit the expression of MRP5, thereby improving the colorectal cancer cells’ sensitivity to oxaliplatin (both *in vivo* and *in vitro*) (Liu et al., 2019).

Tumor-derived exosomes have been shown to be able to target tumors, and exosomes can be used to directly package anticancer drugs and deliver them specifically to gastric and colorectal tumors to inhibit tumor development. They can also use regulatory mechanisms to inhibit the growth of cancer cells or reverse drug resistance by deliver biomolecules such as siRNA.

## Discussion

Gastric and colorectal cancer are common and significant cancers worldwide that cause high morbidity and mortality rate (Athauda et al., 2019). Therefore, it is urgent to develop new diagnostic and therapeutic methods. Increasing numbers of studies indicate that exosomes are involved in various stages of cancer progression and can influence its overall course (Bebelman et al., 2018). For example, exosomes regulate tumor progression due to their ability to communicate between cells and deliver various substances. The contents related to exosomes and cancer regulation introduced in this review mainly include ncRNAs (miRNA, lncRNA, circRNA, piRNA) and proteins, and the role of exosomes includes regulating tumor microenvironments and affecting tumor proliferation, metastasis, and drug resistance (Mashouri et al., 2019). Exosomes secreted by different cells also have different functions and characteristics. In the case of gastric and colorectal cancer cells, they have been shown to promote the proliferation and invasion of their own cancer cells through exosomes and to make the surrounding environment more suitable for tumor growth. Moreover, exosomes secreted by tumor cells can be better ingested by the same type of tumor cells, which indicates that they can be used to carry treatment drugs. In addition, other cells in the tumor microenvironment, such as CAFs, TAMs, and MSCs, have a bidirectional molecular transfer process with tumor cells. In some cases, these cells can promote tumor growth, while in other cases they can deliver substances that inhibit tumor growth. This suggests that the tumor-inhibiting effects can be produced by regulating the tumor microenvironment.

Depending on exosome specificity, exosomes can selectively deliver drugs to specific tumor cells with the advantages of high efficiency and low toxicity (Wu et al., 2021). However, only a few studies have examined the application of exosomal delivery drugs for gastric cancer and colorectal cancer. Moreover, exosomes are rich in miRNAs, which can alter the fate of tumor cells by influencing the expression of related miRNAs in tumor cells (Sun Z. et al., 2018). Studies have found new types of RNA, such as piRNA and tsRNA, in addition to miRNA, lncRNA, and cicrRNA. There have only been a few investigations into the existence and function of these types of ncRNA in gastric cancer and colorectal cancer exosomes. Therefore, although the treatment of exosomes has shown great application prospects in gastric and colorectal cancers, many challenges remain before we can use exosomes in the clinical treatment of cancer. For example, information is still needed regarding the detailed mechanisms of exosomes in cancer cells, the isolation of a large number of exosomes, their precise detection, and the drug loading efficiency and preservation methods of exosomes. In an attempt to improve the safety and quality of exosome engineering in the clinical application of treatments for both gastric and colorectal cancer, it is imperative that further studies be carried out to determine how to improve drug delivery methods and the targeting effect of exosomes.

## Conclusion

Exosomes play an important role in the occurrence and development of gastric and colorectal cancer, and substances such as miRNA that are contained in exosomes can affect tumor progression. Due to their biological characteristics, exosomes can be used for targeted transport of tumor drugs. Therefore, it is reasonable to presume that exosomes can be modified for use as biomarkers, vaccines, and drug carriers to develop more effective clinical diagnosis and treatment strategies for cancer.
